# ACE2 Expression in Kidney and Testis May Cause Kidney and Testis Infection in COVID-19 Patients

**DOI:** 10.3389/fmed.2020.563893

**Published:** 2021-01-13

**Authors:** Caibin Fan, Wei Lu, Kai Li, Yanhong Ding, Jianqing Wang

**Affiliations:** ^1^Department of Urology, The Affiliated Suzhou Hospital of Nanjing Medical University, Nanjing, China; ^2^School of Nursing, Suzhou Vocational Health College, Suzhou, China

**Keywords:** COVID-19, SARS-Cov-2, kidney, testis, ACE2

## Abstract

In December 2019, a new type of pneumonia caused by SARS-Cov-2 (COVID-19) occurred in Wuhan and has been discovered in many countries around the world. ACE2 (angiotensin-converting enzyme 2) has been shown to be one of the major receptors that mediate the entry of SARS-Cov-2 into human cells. Here in this study, we used the online datasets to analyze ACE2 expression in different human organs. The results indicated that ACE2 highly expresses in renal tubular cells, Sertoli cells, Leydig cells, and cells in seminiferous ducts in testis. Recombinant SARS-CoV-2 spike protein (RBD) domain and ACE2 of RPTEC/SerC cell-binding assays confirmed that SARS-Cov-2 can bind to ACE2 on the surface of these cells. Our results suggest that ACE2 expression could contribute to kidney and testis infection after COVID-19 infection. Renal function evaluation and special care should be performed during clinical work. Clinicians should also pay attention to the risk of testicular lesions in patients during hospitalization and later clinical follow-up, especially the assessment and appropriate intervention in young patients' fertility.

## Introduction

Since December 2019, the type of pneumonia caused by the 2019 novel coronavirus disease (COVID-19) was first reported in Wuhan, China, and has been discovered in many countries around the world (1, 2). The virus is a previously unknown sub-coronal virus (β-round virus) named SARS-Cov-2 (severe acute respiratory syndrome coronavirus 2, previously known as 2019-nCoV) by WHO, which forms a branch in the subgenus sarbecovirus, subfamily Orthocoronavirinae (3). SARS-Cov-2 is closely related to SARS-Cov with above 85% identity (4). In addition to common respiratory symptoms such as cough and fever, some patients may also experience other symptoms such as diarrhea and liver damage (5), which brings more challenges to the patient's recovery. Although the source of the SARS-Cov-2 is still unknown, previous research has shown that the receptor-binding domain of SARS-Cov-2 was able to bind ACE2 protein on the surface of human cells, which provide strong evidence for human ACE2 being the receptor for SARS-Cov-2 (6, 7).

ACE2 belongs to the angiotensin-converting enzyme family of dipeptidyl carboxydipeptidases, which is homologous to human angiotensin 1-converting enzyme. The expression distribution of ACE2 suggests that it might play critical roles in the regulation of cardiovascular and renal function, as well as fertility (8). Since the global outbreak of SARS in 2003, numerous studies have revealed the role of cell surface ACE2 as the cellular receptor for SARS-Cov and NL63 (9–11). ACE2 has also been proven to be a major receptor of the novel SARS-Cov-2 because of the close relation to SARS-Cov. As the virus enters the cell by binding to cell receptors to complete intracellular replication, virus release, and induced cytotoxicity, the route of virus infection depends on the expression and distribution of the corresponding receptor (12–14). Meanwhile, the damage caused by the virus in different organs is closely related to clinical manifestations and has a major implication for understanding the pathogenesis and designing therapeutic strategies in clinical practice.

Here in this study, we analyzed the online datasets to uncover the expression pattern of ACE2 in urinary and male reproductive systems, which is the potential mechanism of testis damage, abnormal renal function, or even kidney damage in patients infected with SARS-Cov-2. *In vitro* cell experiments confirmed the possibility of the virus-binding cells with ACE2. Moreover, we emphasized the high ACE2 expression level in testis because of the potential pathogenicity of the virus to testicular tissues, especially the potential risks affecting fertility.

## Materials and Methods

### Clinical Data

We summarized the clinical data of three previous studies to extract the incidence of abnormal renal function or kidney injury in patients with COVID-19 (2, 15, 16). All clinical data are directly obtained from the articles above and we summarized related data in the Supplementary Table 1.

### Publicly Available Gene Expression Data Sets

We used RNA and protein expression data of ACE2 in different human tissues and cancer cell lines through The Human Protein Atlas portal (website: http://www.proteinatlas.org/) (17), GTEx portal (website: https://gtexportal.org,) and The Cancer Cell Line Encyclopedia (CCLE) (18). All data are available directly online.

### Analysis of scRNA-Seq Data

In this article, we made use of some online renal and testicular single-cell RNA-seq (scRNA-seq) gene expression data sets that were publicly usable. These contained the data reported in GSE131685 (for kidney) and GSE112013 (for testis).

Raw reads were processed to generate gene expression matrices as described previously (19, 20). CellRanger (Version: 3.0.1, 10X Genomics) processing was used to compare the sequencing results of each sample to the GRCh38 reference genome. The gene expression matrix was generated in R (Version: 3.6.1) using the Seurat R package (Version: 3.1.3) The Read10X function loads and constructs a Seurat object based on each experiment. After filtering <500 gene cells and mitochondrial expression rates of more than 20%, we then used Seurat's CCA algorithm to perform data reconciliation and correct batch effects between data to reduce differences between technical iterations. Then, we performed t-SNE for dimensionality reduction and cluster analysis using the first 2000 high variable genes of the integer and post-Seurat. We calculated the gene specifically expressed by each cluster through the FindAllMarker function and annotated the cell type based on the specifically expressed gene. All cell markers were shown as follows: GSE131685 (for kidney): proximal convoluted tubule cells (GPX3), proximal tubule cells (DCXR), proximal straight tubule cells (SLC22A7), NK-T cells (CD3E), monocytes (CD14), glomerular parietal epithelial cells (KRT8), distal tubule cells (DEFB1), collecting duct principal cells (AQP2), B cells (CD79A), and collecting duct intercalated cells (ATP6V1G3).

GSE112013 (for testis): SSCs (ID4, UTF1, FGFR3), differentiating S'gonia (KIT), early primary S'cytes (MAGEA4, DAZL, SYCP3, DMRTB1), late primary S'cytes (SPO11, MLH3, ZPBP), round S'tids (ZPBP), elongated S'tids (TNP1, PRM2, ZPBP, ZQTN), sperm (TNP1, PRM2, FAM71B), sperm (TNP1, PRM2, LELP1, PRM2), macrophage (VIM, CD14), endothelial cells (VIM, VWF), myoid cells (VIM, ACTA2), Sertoli cells (VIM, GSTA1, BEX1, APOA1), Leydig cells (VIM, DLK1).

### Cell Lines and Cell Culture

Human Sertoli cells (HserC, Cat. #4520) were obtained from ScienCell through Shanghai Zhong Qiao Xin Zhou Biotechnology Co., Ltd. and maintained in Sertoli cell medium (SerCM, Cat. #4521) supplemented with 10% FBS and antibiotics (100 units/ml penicillin and 0.1 mg/ml streptomycin). RPTEC (renal proximal tubular epithelial cells) was obtained from ATCC and maintained in RPMI-1640 medium (Gibco) supplemented with 10% FBS and antibiotics. All cells were grown at 37°C in standard cell culture conditions (5% CO2, 95% humidity). In the culture process, all cells were passaged every 2–3 days by mild trypsinization in 10-cm dishes. Fresh medium was provided 2–3 times per week. All cell lines were authenticated by STR profiling and tested for mycoplasma contamination.

### SARS-CoV-2 RBD Domain and ACE2 of RPTEC/SerC Cell Binding Assays

#### SARS-CoV-2 RBD Biotin Labeling

First of all, we calculated the amount of biotin reagent added according to 20 mol biotin per mol protein. For example: 1 ml 0.3 mg/ml SARS-CoV-2 RBD protein requires biotin reagent amount:

- $\begin{array}{l} 1ml\;X\;\times \;\frac{0.3mg\;X}{1\;ml\;X}\;\times \;\frac{1mmol\;X}{70,000mg\;X}\;\times \;\frac{20mmol\;Biotin}{1mmol\;X}=0.000086\text{mmol}\tilde{\text{B}}\text{iotin} \end{array}$
- $\begin{array}{l} 0.000086mmol\;Biotin\;\times \frac{1,000,000\;ul}{L}\;\times \frac{L}{10\;mmol}=8.6\;ul\;Biotin\;Reagent \end{array}$

Biotin reagent was prepared by adding 180 μl of DMSO to the powder to obtain 10 mM biotin reagent. 8.6 μl biotin reagent was added to 0.3 mg/ml SARS-CoV-2 RBD protein. Then, a desalting process was performed through a desalting column following the Thermo protocol, and the concentration of protein was measured. Finally, we detected the protein activity by ELISA.

#### Staining and Flow Cytometry

For flow cytometry, we divide 10^6^/well cells into the flow tube with 10 μg/ml biotin–SARS-CoV-2 RBD. Staining buffer (2% FBS in DPBS) was used to remove unbound biotin–SARS-CoV-2 RBD to prevent non-specific binding. BV421–streptomycin was used as the secondary antibody. Cells were detected using pacific blue in flow cytometry (BD Fortessa).

#### Syncytia Formation Assay

A PCMV-2019nCOV full-length plasmid or control vector was transferred into 293T cells using Lipofectamine 2000 transfection reagent (Invitrogen). Twenty four hours after transfection, cells were cultured in the normal medium for 24 h. After that, 1 × 10^5^ digested 293T cells were cocultured with 1 × 10^5^ RPTEC cells for 48 hours. Pictures of syncytia formation were then taken with a 20 × bright-field fluorescence inverted microscope.

### Statistical Analyses

The results were presented as the average values ± standard error of mean (SEM). ACE2 expression levels in different cell types were evaluated by the use of Student's *t*-test. A p value below 0.05 was thought to have statistical significance, ^*^P < 0.05, ^**^P < 0.01, and ^***^P < 0.001. All the statistical analyses were conducted with GraphPad and R 3.6.1.

## Results

### Abnormal Renal Function or Kidney Injury in COVID-19 Patients

At first, we tried to investigate whether abnormal renal function or kidney injury happened in COVID-19 patients. Since the number of patients in our region is relatively small, we reviewed 3 studies focused on the clinical features of such patients and summarized related data. In these 3 cohorts, one was a familial cluster of six patients [cohort 3 in Supplementary Table 1, (2)], while other cohorts contained 99 patients [cohort 1 in Supplementary Table 1, (15)] and 41 patients [cohort 2 in Supplementary Table 1, (16)], respectively. Results suggested that about 3–10% of COVID-19 patients had abnormal renal function, including elevated creatinine or urea nitrogen. In addition, 7% of patients experienced acute renal injury (Supplementary Table 1). Moreover, a latest study also indicated that 10.8 and 7.2% patients also showed mild elevation of blood urea nitrogen or creatinine, and trace or 1+ albuminuria, respectively, in 111 COVID-19 patients without basic kidney disease in a single hospital in Wuhan, while another study showed that 5 in 138 COVID-19 patients had acute kidney injury [data not shown, (21, 22)]. All these results indicated that abnormal renal function or kidney injury happened in a considerable number of COVID-19 patients. Considering the large number of infected patients, it is necessary to explore the mechanisms of abnormal renal function and to promote to take special care on such patients.

### ACE2 Expresses Highly in Renal and Testicular Cells

As the virus frequently enters the cell by binding to cell receptors, and ACE2 has been proven to be one of the major receptors of SARS-Cov-2 in the human body, we explored the online datasets to find out the expression level of ACE2 in the urinary system. As we expected, data from the CCLE and GTEx portal indicated that the ACE2 mRNA expression level is relatively higher in kidney cells (Supplementary Figures 1A, 2).

To further determine the protein expression level of ACE2 in renal cells, we investigated the Human Protein Atlas portal to find out some details. Results of immunohistochemistry (IHC) also indicated that the expression level of ACE2 protein is significantly higher in the kidney, especially in renal tubular cells, although the mRNA expression level is not such high (Supplementary Figures 1B,C, 3 and Table 1).

**Table 1:** Summary of the IHC results in the human protein atlas project.

**Testis (*****n*** **=** **6)**				**Kidney (*****n*** **=** **6)**			
**Cell type**	**Characteristics**	**IHC results**		**Cell type**	**Characteristics**	**IHC results**	
Cells in seminiferous ducts	Staining:	High	6/6 (100%)	Cells in glomeruli	Staining:	Not detected	6/6 (100%)
	Intensity:	Strong			Intensity:	Negative	
	Quantity:	>75%			Quantity:	None	
Leydig cells	Staining:	High	6/6 (100%)	Cells in tubules	Staining:	High	6/6 (100%)
	Intensity:	Strong			Intensity:	Strong	
	Quantity:	>75%			Quantity:	75–25%	

In addition, we also found that ACE2 expresses quite highly in testicular cells. The protein and mRNA expression of ACE2 in the testes is almost the highest in the body. Moreover, both cells in seminiferous ducts and Leydig cells showed a high ACE2 expression level (Supplementary Figures 1B,D, 3 and Table 1). These results indicate that testicular cells are the potential targets of SARS-Cov-2.

### Results of scRNA-seq Data of Kidney and Testis

**Figure 1:**
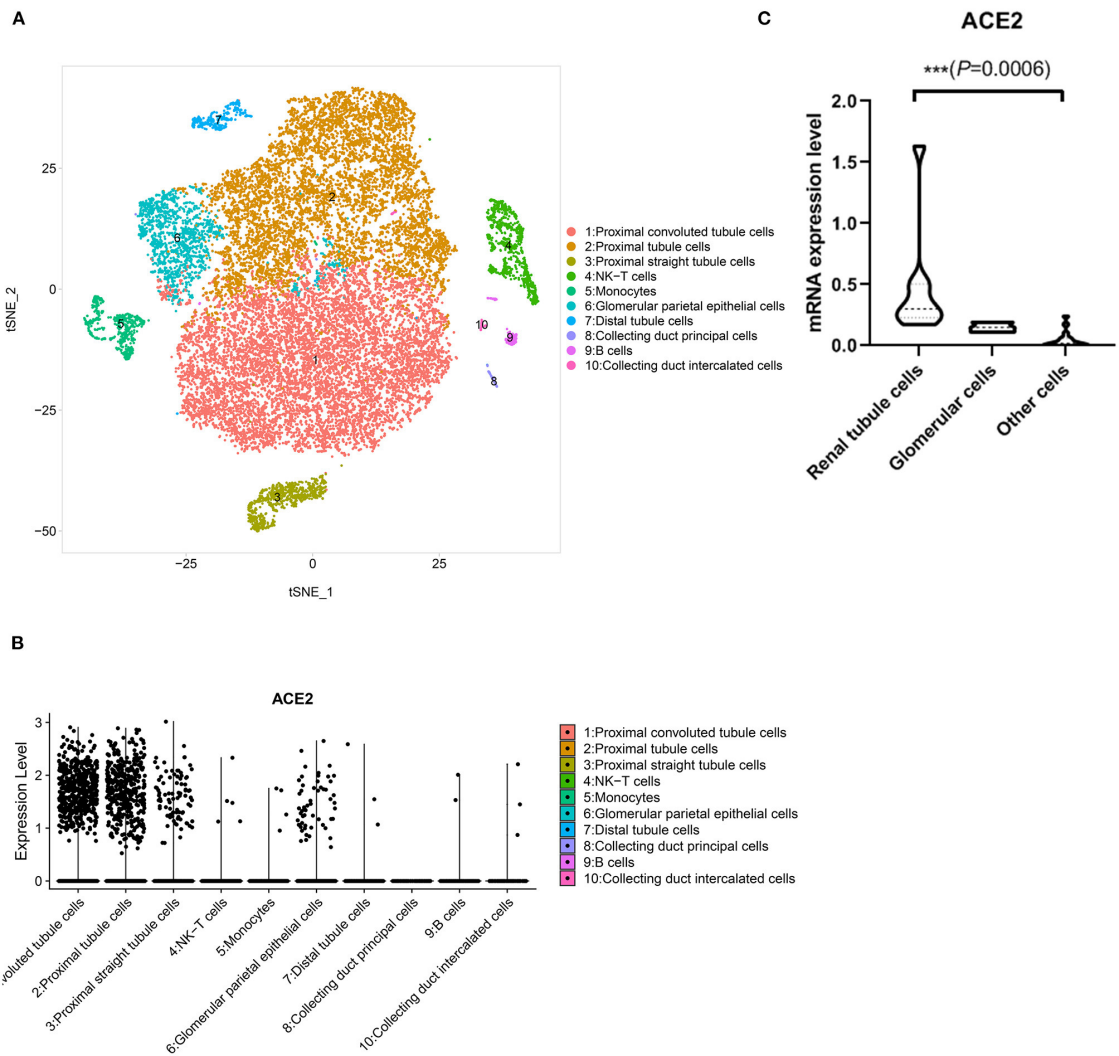
Single-cell analysis of published renal cell atlas. **(A)** Renal cell atlas visualized by UMAP, colored by cluster number. Cluster number information is provided by the authors. **(B)** The dotplot showing ACE2 gene expression of all major cell types. **(C)** Violin plots of ACE2 expression in renal cells and other cells.

To further assess the cell type-specific expression of ACE2 and confirm the ACE2 expression level in kidney and testis, we downloaded the gene expression data of single-cell RNA sequencing of human kidney and testis from Gene Expression Omnibus (GEO) datasets. We first analyzed GSE131685, a published dataset containing scRNA-seq data of the normal kidney samples from 3 donors (19). We divided single cells into subclusters based on the canonical markers and cell classification in the original literature (Figure 1A) and found specific ACE2 expressions in tubular cells. In contrast, ACE2 expression was not observed in immune cells and glomerular parietal epithelial cells (Figures 1B,C).

**Figure 2:**
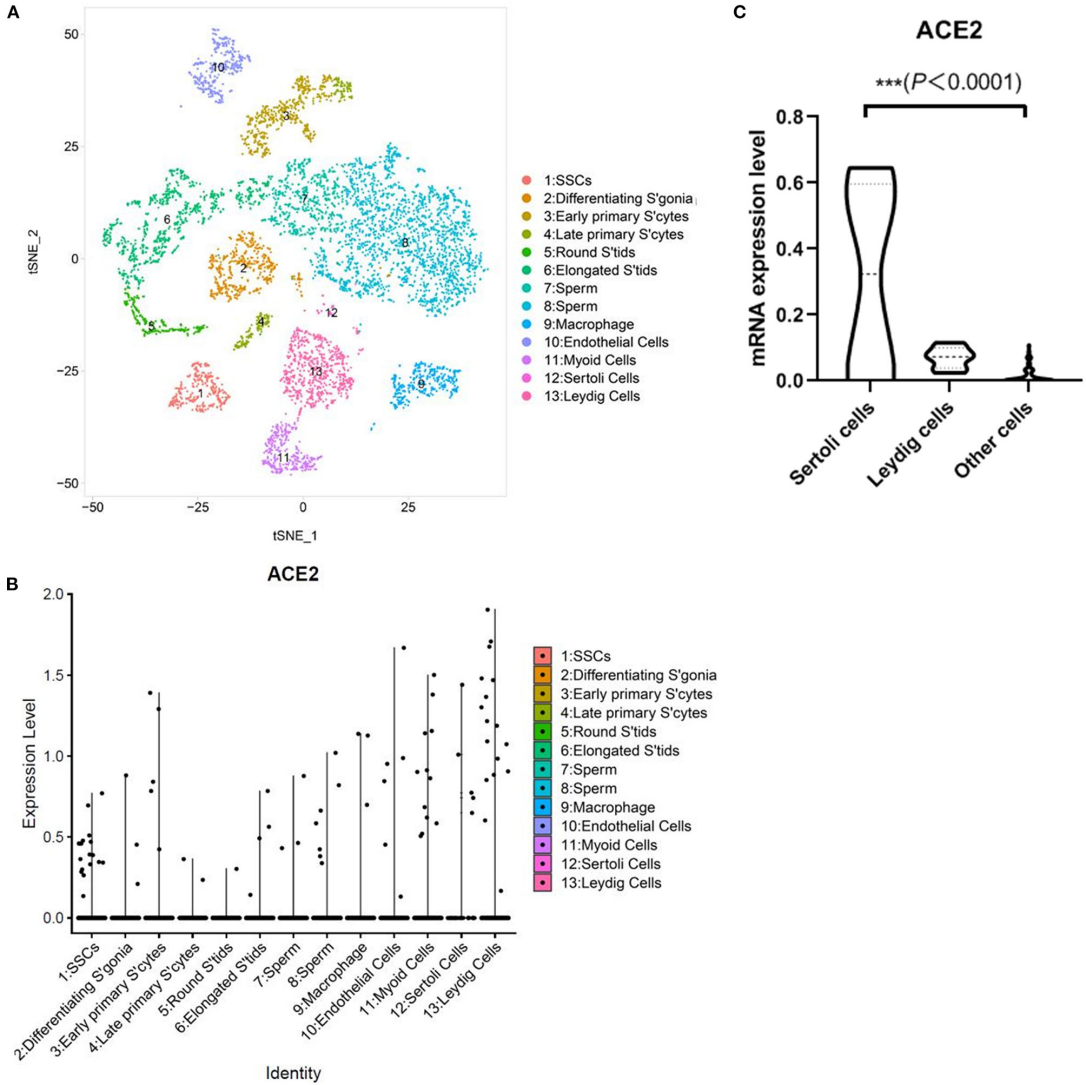
Single-cell analysis of published testicular cell atlas. **(A)** Testicular cell atlas visualized by UMAP, colored by cluster number. Cluster number information is provided by the authors. **(B)** The dotplot showing ACE2 gene expression of all major cell types. **(C)** Violin plots of ACE2 expression in Sertoli cells, Leydig cells, and other cells.

Then, we downloaded the data of GSE112013, an open dataset containing scRNA-seq data of the normal human adult testis samples from 3 donors (20). We divided single cells into 13 subclusters based on the canonical markers and cell classification in the original literature (Figure 2A). Results indicated the high expression levels of ACE2 in Sertoli cells and Leydig cells, especially in Sertoli cells, compared with other cell types (Figures 2B,C).

Therefore, ACE2 expression in renal tubular cells and testicular cells (mainly in Sertoli cells and Leydig cells) may suggest a potential mechanism of infection and direct injury of renal tubules and testis by SARS-Cov-2 binding ACE2 as host cell receptors.

### Results of SARS-Cov-2 RBD Domain and ACE2 of RPTEC/SerC Cell-Binding Assays

**Figure 3:**
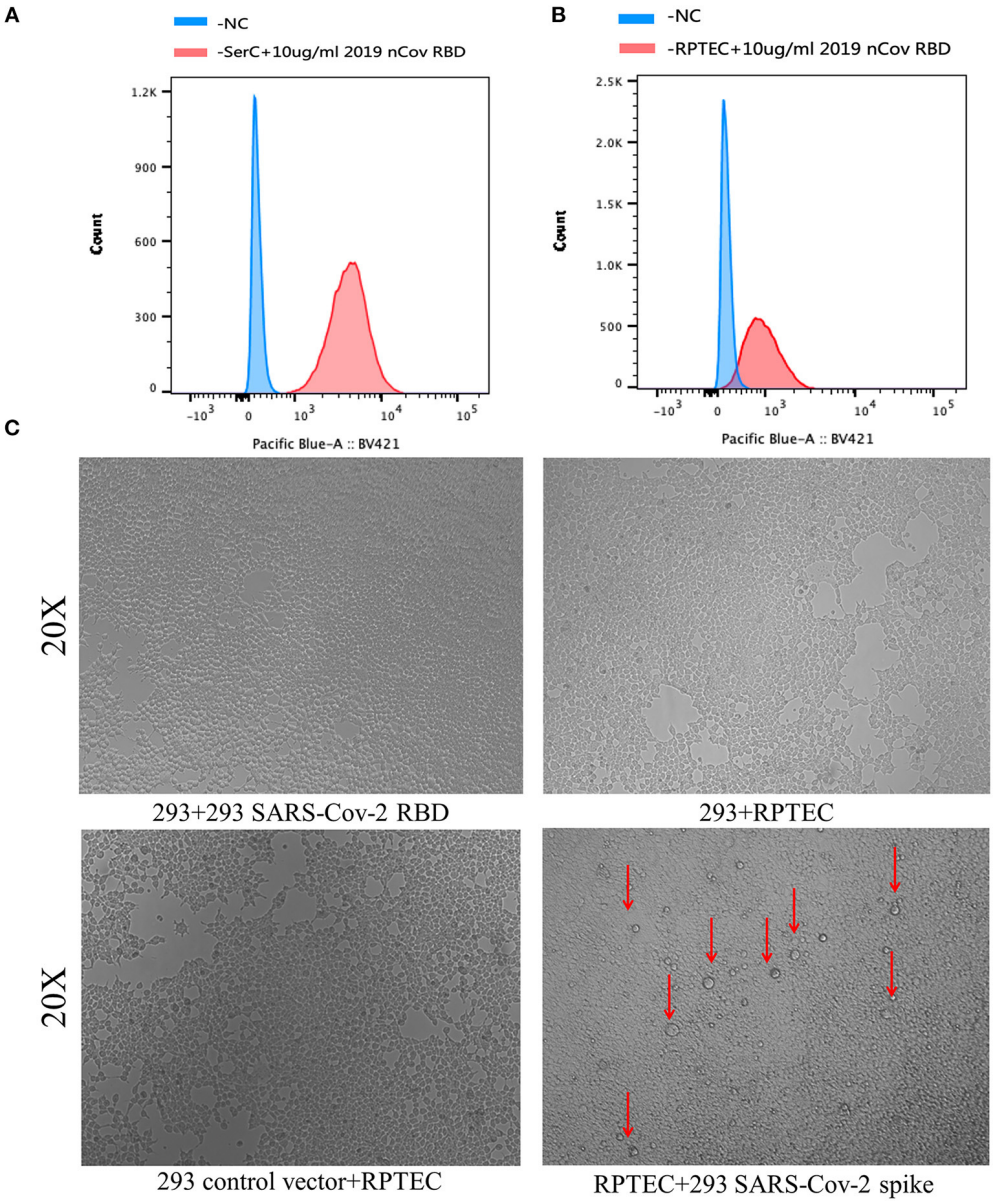
Binding assays of SARS-Cov-2 RBD domain and ACE2 of RPTEC/SerC cells. **(A)** The positive peak of the horizontal axis showed the significant binding of the SARS-Cov-2 RBD domain to ACE2 of SerC cells. **(B)** The positive peak of the horizontal axis showed the significant binding of the SARS-Cov-2 RBD domain to ACE2 of RPTEC cells. **(C)** Result of syncytia formation experiment of RPTEC cells and 293 cells with SARS-Cov-2 RBD overexpression showed the binding of SARS-Cov-2 RBD to ACE2 on the surface of RPTEC cells. Red arrows indicate part of the representative syncytia formation.

To further confirm whether SARS-Cov-2 could bind ACE2 of renal tubular cells and testicular cells to infect or enter the host cells, we did an *in vitro* mimic experiment to examine the binding of the SARS-Cov-2 RBD domain and RPTEC/SerC cells. As shown in Figure 3A, SARS-CoV-2 RBD can strongly bind to ACE2 on the surface of Sertoli cells, which indicates that SARS-CoV-2 can easily infect these male germ cells in testis.

Then, we did the same assay in RPTEC cells, a kind of renal proximal tubular epithelial cell. Results showed that SARS-Cov-2 RBD can also strongly bind to ACE2 on the surface of RPTEC cells, which is consistent with the results of bioinformatic analysis, even though the binding to RPTEC is weaker than that in Sertoli cells (Figure 3B). This may be due to the different expression level and affinity of ACE2 receptors on the two cell surfaces. Moreover, syncytial formation assay further confirmed the result in RPTEC cells, suggesting the infection of SARS-CoV-2 to renal tubular cells (Figure 3C).

## Discussion

Novel coronavirus-infected pneumonia COVID-19 broke out in Wuhan in December 2019 and January 2020, which has posed a major threat to global public health (23). The numbers of confirmed cases and deaths are still rising quickly all around the world, posing higher challenges for disease control and patient treatment. The symptoms of the disease are complex. In addition to common respiratory symptoms such as cough and fever, some patients may also experience other symptoms such as diarrhea and liver damage (5), or even asymptomatic, which brings more challenges to the patient's diagnosis and treatment. Studies on the mechanisms of disease pathogenesis could help us better understand the disease comprehensively.

Previous literatures showed that about 10% of the COVID-19 patients had abnormal renal function. Although the proportion of patients with abnormal renal function is not so high, and some researchers suggested that SARS-Cov-2 infection does not significantly cause acute renal injury (21), abnormal renal function should still be noted due to the current large number of infections. Here in this study, we used the online datasets and bioinformatic methods and found out that ACE2, one of the major receptors for SARS-Cov-2, expresses quite highly in renal cells, particularly in tubular cells. The SARS-Cov-2 RBD domain and ACE2 of RPTEC cell-binding assays confirmed that SARS-Cov-2 could successfully infect ACE2 positive renal tubular cells.

Under physiological conditions, renal tubular cells have reabsorption and excretion functions and play a key role in excretion of metabolites and maintenance of body fluid balance and acid–base balance. Renal tubular cell injury could cause renal tubules atrophy, thereby aggravating renal interstitial fibrosis, secreting a variety of chemokines and growth factors into the stroma, promoting interstitial inflammatory cell infiltration, interstitial intrinsic cell proliferation, and extracellular matrix (ECM) accumulation. Therefore, SARS-Cov-2 could enter the renal tubular cell by binding to ACE2 and induce cytotoxicity and abnormal renal function, which is a potential mechanism how abnormal renal function happens in COVID-19 patients. Besides our findings, some preliminary results of other research groups have also confirmed our results. A latest online preprint illustrated that kidney tissues from postmortems showed severe acute tubular necrosis and lymphocyte infiltration, and SARS-Cov-2 NP antigen was accumulated in kidney tubules, which is consistent with our findings (24). SARS-Cov-2 RNA was found to be positive in urine sediments in some patients without renal illness before (25), which confirms that the virus can enter the kidney cells through the blood and then into the urine. Examination and follow-up of the renal function of COVID-19 patients are necessary to detect the impaired renal function in time and provide early intervention.

Another major point in this study is the potential infection of SARS-Cov-2 in testicular cells. It is well-known that viruses such as HIV, HBV, and mumps could enter the testicular cells and cause viral orchitis. Besides, in some cases, virus-induced testicular tissue damage might result in male infertility and testicular tumor (26). SARS-Cov is just like the “cousin” of SARS-Cov-2 and shares the receptor ACE2 with SARS-CoV-2. Previous research has investigated the possible damage of the testis in SARS patients and the effects of SARS-Cov on spermatogenesis. Their findings suggested that orchitis is a complication of SARS and that spermatogenesis could be affected after infection (27). Our results showed the high expression level of ACE2 in testicular cells, especially in Sertoli cells and Leydig cells. *In vitro* assays also showed the binding of SARS-Cov-2 to Sertoli cells. Thus, one potential direct mechanism how the virus damages the testis is that SARS-Cov-2 could bind and enter such ACE2-positive cells. Current clinical data show that a large proportion of COVID-19 patients are young adults and even children, so the potential testicular damage caused by the virus may exist as a late complication.

Another potential indirect mechanism might be the destruction of the blood–testis barrier. As we all know, a fortress created by the blood–testis barrier could provide an optimal environment for the survival of germ cells by prevent or limit the immune response (28, 29). Sertoli cells (SCs), which are ACE2 highly expressed, protect germ cells by forming the blood–testis barrier and regulating testicular milieu (30). SARS-Cov-2 could infect Sertoli cells and damage the blood–testis barrier by binding ACE2, other than directly entering cells or testis tissues, resulting in leukocyte infiltration, which also happened in SARS. Research on SARS indicated the orchitis without SARS-Cov positivity in testis tissues (27). These cells may then affect the function of Leydig cells and testosterone production and directly damage the seminiferous epithelium. Finally, these cells and their products, inflammatory cytokines, may activate autoimmune responses and autoantibody development in the tubules.

Another major significance of our research is to attract the attention of doctors, patients, and researchers. It is hoped that doctors could follow up patients appropriately to monitor changes in renal function, especially fertility in young male patients. At the same time, patients with orchitis as the first symptom should be noticed and rule out SARS-Cov-2 infection. It is hoped that patients could pay attention to the symptoms caused by COVID-19 in other organs and cooperate with doctors for follow-up and treatment. It is hoped that qualified researchers could focus on testicular lesions during anatomy, deepen research and cooperation, and advance research on the mechanism and treatment of the disease.

Our research contains some limitations. There is still limited information available on the impact of SARS-Cov-2 on the reproductive system because of the lack of anatomy and pathology, which is also the limitation of our study. One recent online preprint provided the evidence about the influence of COVID-19 on male sex hormones, which could validate the damage of SARS-Cov-2 on the testis to a certain extent (31). Due to the differences between the two cells in cell culture and the experimental conditions, we were unable to complete syncytia formation experiments in Sertoli cells, or to perform virus invasion experiments, which is another limitation. Ultimately, whether SARS-Cov-2 can cause testis damage and what the possible damage mechanism is still depends on the results of anatomy and pathology. Our findings suggest the significance of the anatomical and pathological study on testes of COVID-19 patients. Clinicians should take care of the possible occurrence of orchitis. Follow-up and evaluation of the reproductive functions should be done in recovered male COVID-19 patients, especially the young male patients.

## Conclusions

Our study demonstrated the high expression of ACE2 in kidney and testicular tissue and showed possible mechanisms of SARS-Cov-2 infecting such cells by binding ACE2. Our results facilitate the understanding of the mechanisms of abnormal renal function and kidney damage in COVID-19 patients and suggest that the patient cares regarding the possible occurrence of orchitis. Follow-up and evaluation of the reproductive functions may be necessary in recovered male COVID-19 patients, especially the young male patients.

## Data Availability Statement

The original contributions presented in the study are included in the article/Supplementary Material, further inquiries can be directed to the corresponding author/s.

## Author Contributions

JW: conceptualization, methodology, writing- reviewing and editing, funding acquisition. CF: data curation, methodology, writing- original draft preparation. WL: data acquisition, investigation. KL and YD: software, validation. All authors: contributed to the article and approved the submitted version.

## Conflict of Interest

The authors declare that the research was conducted in the absence of any commercial or financial relationships that could be construed as a potential conflict of interest.

## Supplementary Material

Supplementary Figure 1 Data of expression level of ACE2 in different human tissues from online datasets. **(A)** Data from CCLE showed ACE2 expression level in different tissues, including urinary system (red frame). **(B)** ACE2 protein expression level in different tissues. **(C,D)** Representative IHC staining of ACE2 in kidney and testis.

Supplementary Figure 2 GTEx portal showed ACE2 expression level in different tissues. The expression levels of renal and testicular cells were indicated using # or ^*^.

Supplementary Figure 3 Data of mRNA or protein expression level of ACE2 in different human tissues from HPA online datasets.
