# Supplementary material for: ACE2 Expression in Kidney and Testis May Cause Kidney and Testis Infection in COVID-19 Patients
Source: Front Med (Lausanne). 2021 Jan 13;7:563893. doi: 10.3389/fmed.2020.563893 (PMC7838217; doi:10.3389/fmed.2020.563893)
Supplement: Supplementary file 1 [file Table_2.DOCX]

| Supplementary Table 1. Summary of the renal function characteristics of patients infected with 2019-nCoV in 3 cohorts | | | | | |
| --- | --- | --- | --- | --- | --- |
| Characteristics | Cohort 1 |  | Cohort 2 |  | Cohort 3 |
| **Patients** | n=99 |  | n=41 |  | n=6 |
| **Age, years [median (range)]** | 55.5 (21-82) |  | 49 (41–58) |  | 36.5 (10-66) |
| **Sex** |  |  |  |  |  |
| Female | 32 (32%) |  | 11 (27%) |  | 3 |
| Male | 67 (68%) |  | 30 (73%) |  | 3 |
| **Renal Function** |  |  |  |  |  |
| **Blood urea nitrogen ^#^** |  |  |  |  |  |
| Increased | 6 (6%) |  | N/A |  | 0 |
| **Serum creatinine ^##^** |  |  |  |  |  |
| Increased | 3 (3%) |  | 4/41 (10%) |  | 2 |
| **Other renal related affairs** |  |  |  |  |  |
| Acute kidney injury **^###^** | N/A |  | 3 (7%) |  | N/A |

# In cohort 1: Blood urea nitrogen (mmol/L; normal range 3.6–9.5)

## In cohort 1: Serum creatinine (μmol/L; normal range57.0–111.0); In cohort 2: Serum creatinine (μmol/L; normal ≤133)

### Acute kidney injury was defined on the basis of the highest serum creatinine level or urine output criteria according to the kidney disease improving global outcomes classification ([16](#_ENREF_16)).
