# Supplementary figures and images for: ACE2 Expression in Kidney and Testis May Cause Kidney and Testis Infection in COVID-19 Patients
Source: Front Med (Lausanne). 2021 Jan 13;7:563893. doi: 10.3389/fmed.2020.563893 (PMC7838217; doi:10.3389/fmed.2020.563893)

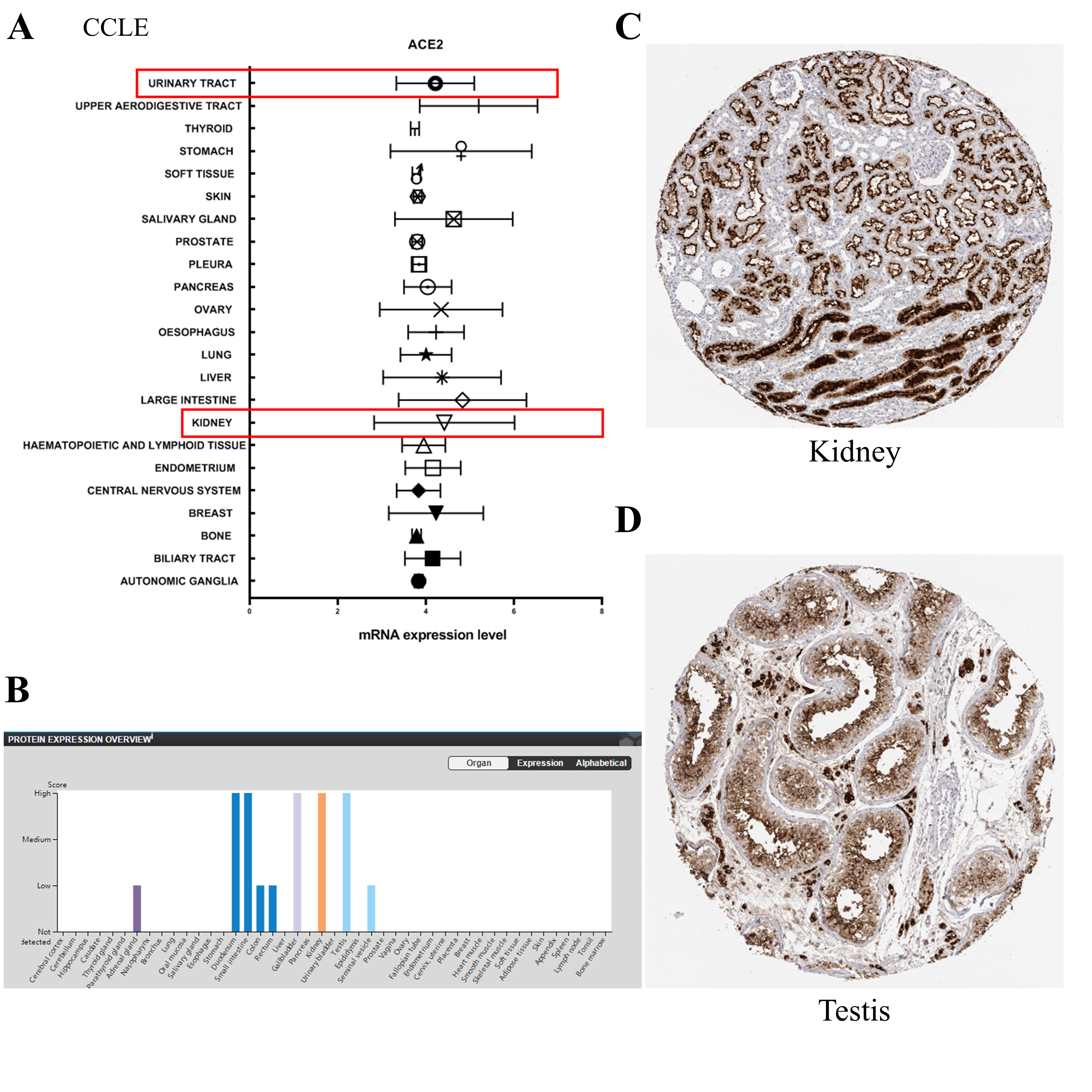

Supplement: Supplementary Figure 1 — Data of expression level of ACE2 in different human tissues from online datasets. (A) Data from CCLE showed ACE2 expression level in different tissues, including urinary system (red frame). (B) ACE2 protein expression level in different tissues. (C,D) Representative IHC staining of ACE2 in kidney and testis. [file Image_1.TIF]

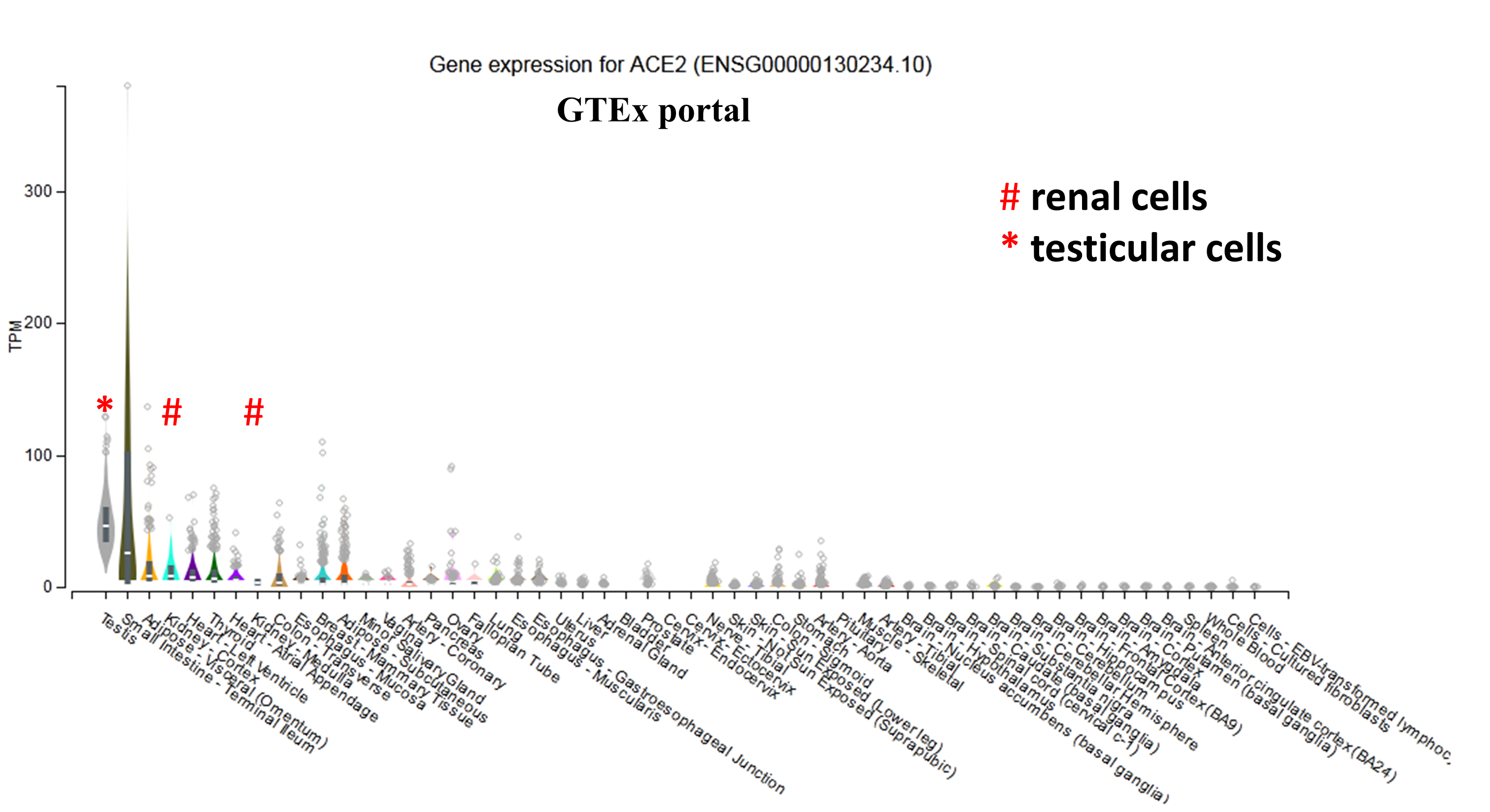

Supplement: Supplementary Figure 2 — GTEx portal showed ACE2 expression level in different tissues. The expression levels of renal and testicular cells were indicated using # or *. [file Image_2.TIF]

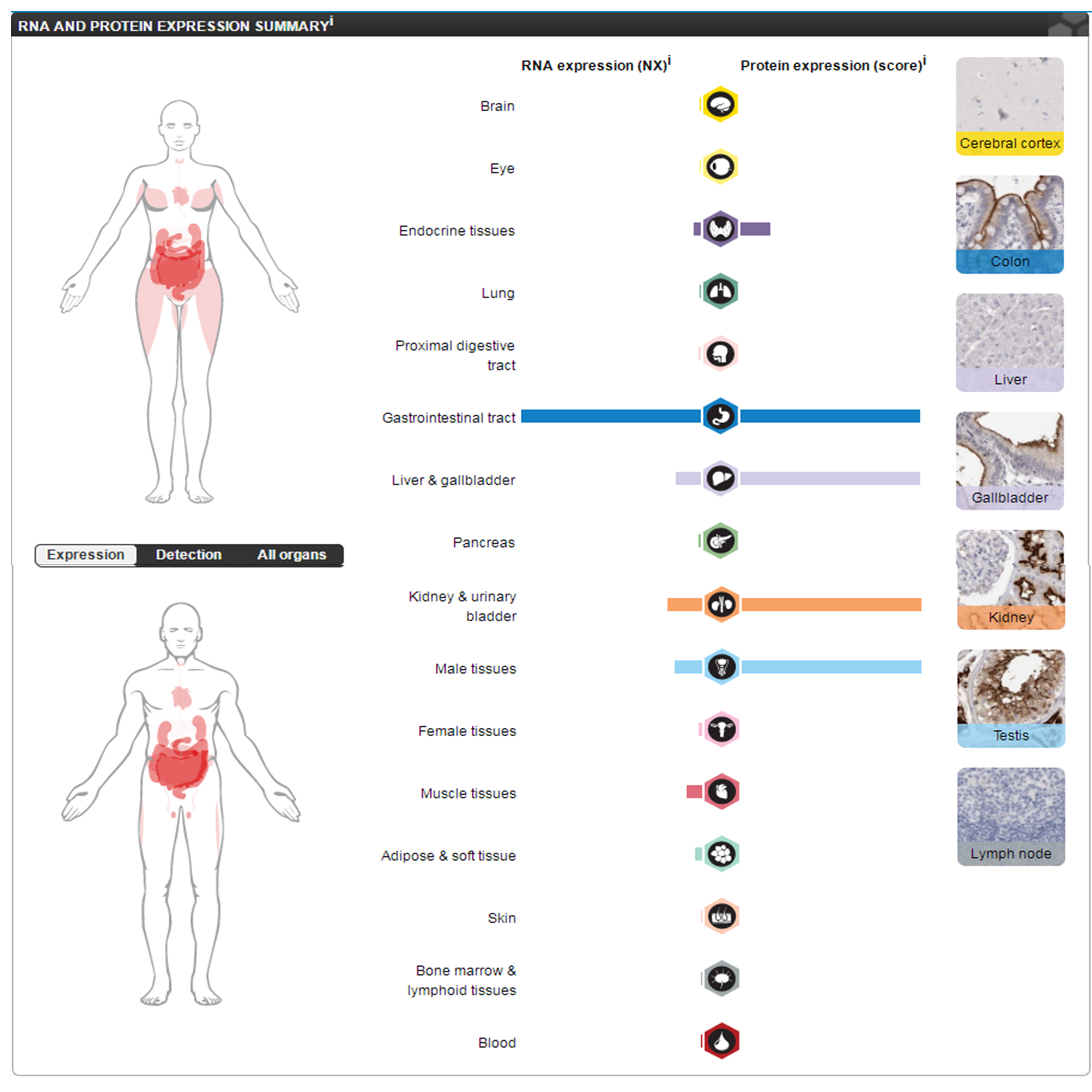

Supplement: Supplementary Figure 3 — Data of mRNA or protein expression level of ACE2 in different human tissues from HPA online datasets. [file Image_3.TIF]
